# Automated 3D segmentation and diameter measurement of the thoracic aorta on non-contrast enhanced CT

**DOI:** 10.1007/s00330-018-5931-z

**Published:** 2019-01-23

**Authors:** Zahra Sedghi Gamechi, Lidia R. Bons, Marco Giordano, Daniel Bos, Ricardo P. J. Budde, Klaus F. Kofoed, Jesper Holst Pedersen, Jolien W. Roos-Hesselink, Marleen de Bruijne

**Affiliations:** 1 Biomedical Imaging Group Rotterdam, Departments of Radiology and Medical Informatics, Erasmus MC, Rotterdam, The Netherlands; 2 Department of Cardiology, Erasmus MC, Rotterdam, The Netherlands; 3 Department of Radiology and Nuclear Medicine, Erasmus MC, Rotterdam, The Netherlands; 4 Department of Epidemiology, Erasmus MC, Rotterdam, The Netherlands; 5 Department of Cardiothoracic Surgery, Copenhagen University Hospital, Rigshospitalet, Copenhagen, Denmark; 6 Machine Learning Section, Department of Computer Science, University of Copenhagen, Copenhagen, Denmark

**Keywords:** Thoracic aorta, Dilatation, Computer-assisted image analysis, Three-dimensional image, Computed tomography, X-ray

## Abstract

**Objectives:**

To develop and evaluate a fully automatic method to measure diameters of the ascending and descending aorta on non-ECG-gated, non-contrast computed tomography (CT) scans.

**Material and methods:**

The method combines multi-atlas registration to obtain seed points, aorta centerline extraction, and an optimal surface segmentation approach to extract the aorta surface around the centerline. From the extracted 3D aorta segmentation, the diameter of the ascending and descending aorta was calculated at cross-sectional slices perpendicular to the extracted centerline, at the level of the pulmonary artery bifurcation, and at 1-cm intervals up to 3 cm above and below this level. Agreement with manual annotations was evaluated by dice similarity coefficient (DSC) for segmentation overlap, mean surface distance (MSD), and intra-class correlation (ICC) of diameters on 100 CT scans from a lung cancer screening trial. Repeatability of the diameter measurements was evaluated on 617 baseline-one year follow-up CT scan pairs.

**Results:**

The agreement between manual and automatic segmentations was good with 0.95 ± 0.01 DSC and 0.56 ± 0.08 mm MSD. ICC between the diameters derived from manual and from automatic segmentations was 0.97, with the per-level ICC ranging from 0.87 to 0.94. An ICC of 0.98 for all measurements and per-level ICC ranging from 0.91 to 0.96 were obtained for repeatability.

**Conclusion:**

This fully automatic method can assess diameters in the thoracic aorta reliably even in non-ECG-gated, non-contrast CT scans. This could be a promising tool to assess aorta dilatation in screening and in clinical practice.

**Key Points:**

*• Fully automatic method to assess thoracic aorta diameters.*

*• High agreement between fully automatic method and manual segmentations.*

*• Method is suitable for non-ECG-gated CT and can therefore be used in screening.*

**Electronic supplementary material:**

The online version of this article (10.1007/s00330-018-5931-z) contains supplementary material, which is available to authorized users.

## Introduction

Aortic aneurysm with the risk of acute dissection is an important cause of mortality in the western world [1]. The prevalence of thoracic aortic aneurysms is estimated around 0.3% in the normal population [2, 3]. Most patients with a dilated aorta or aortic aneurysm are asymptomatic. The diagnosis can be made as during screening in the context of a positive family history or by coincidence on imaging examinations performed for other purposes like lung cancer screening [4]. However, acute dissection is often the first presentation, in which case over 50% of all patients die within 30 days [5].

Because of this silent process with high risks, screening programs using non-contrast computed tomography (CT) could be considered. In patients with aortic aneurysms, the aortic size has a profound impact on the risk of dissection [6, 7]. Detecting aortic dilatation at an early stage enables preventive surgery, which might save lives. CT imaging of the thoracic aorta could become available as part of a comprehensive assessment of CT imaging performed for screening purposes including also other organs (lungs, coronary calcium, vertebral bone density, etc.) [4].

By measuring aortic dimensions in such screening cohorts we will also gain more information on normal values of aortic diameters, normal increase in diameters over time, and risk factors for dilatation, and a better insight in prognosis.

Besides its potential in screening, non-contrast CT is frequently used in diagnosis and follow-up of patients in clinical practice. It plays a central role in the imaging of the thoracic aorta because of the short time required for image acquisition, the ability to obtain a complete 3D view of the entire aorta, and its widespread availability. CT scans can be used for follow-up of patients with dilatation, especially in cases where echocardiography does not adequately visualize the dilatation. The ESC Guidelines and ACCF/AHA guidelines [8, 9] describe standard anatomical landmarks for reporting aortic diameters in CT in clinical practice.

Performing measurements of the aorta manually is labor intensive and subject to inter-observer variability. To assess aortic dilatation both in screening settings and in clinical practice, automated aorta segmentation and subsequent diameter analysis are therefore desirable. While automatic solutions for aortic measurements in CT angiography (CTA) exist [10–14], automatic aorta segmentation in non-contrast CT scans is more challenging due to the lack of contrast between blood pool regions and surrounding soft tissue [15–19].

The aim of the current study is to develop and validate an automatic method to robustly assess diameters of the ascending and descending aorta in non-ECG-gated, non-contrast CT without human interaction.

## Materials and methods

### Study population and image acquisition

The CT scans used in this study are from the Danish Lung Cancer Screening Trial [20]. A multi-detector CT scanner (Mx 8000 IDT 16 row scanner, Philips Medical Systems) was used to acquire CT scans at 120 kV/40 mAs at maximum inspiration breath hold and without cardiac gating. This protocol leads to an effective dose of around 1 mSv [21]. The scans were reconstructed with a sharp kernel (Philips D), in-plane isotropic resolution of 0.78 × 0.78 mm, and 1-mm slice thickness. Participants were current or former smokers between 50 and 70 years of age. For this study, 742 participants were randomly selected, which were divided into three non-overlapping sets (see supplementary Table S1 for clinical characteristics of the entire data):

- Baseline scans of 25 subjects for parameter optimization of the proposed method
- Baseline scans of 100 subjects for evaluation of the method’s accuracy (see Table 1)
- Baseline and first-year follow-up scans of 617 subjects to evaluate the repeatability of the method

**Table 1:** Clinical characteristics of 100 subjects used in validation. Values are expressed as mean ± standard deviation and (range)

Validation set (*n* = 100)	Male	Female
Number of CT scans (*n*)	50	50
Age (years)	58.5 ± 5.4 (50–70)	58.3 ± 4.8 (50–70)
Weight (kg)	84.0 ± 12.0 (60–120)	67.6 ± 12.2 (48–103)
Height (cm)	179.8 ± 6.3 (163–195)	167.0 ± 6.1 (155–179)
BMI	26.0 ± 3.6 (18.7–37.0)	24.3 ± 4.7 (16.2–41.3)
Agatston score at ascending aorta and arch	231.3 ± 416.7 (0–2190)	193.3 ± 274.4 (0–1128)
Agatston score at descending aorta	53.5 ± 116.2 (0–483)	81.4 ± 316.4 (0–2139)

Therefore, aortic diameter measurements were performed in 1334 CT scans in total.

### Manual annotation

**Fig. 1:**
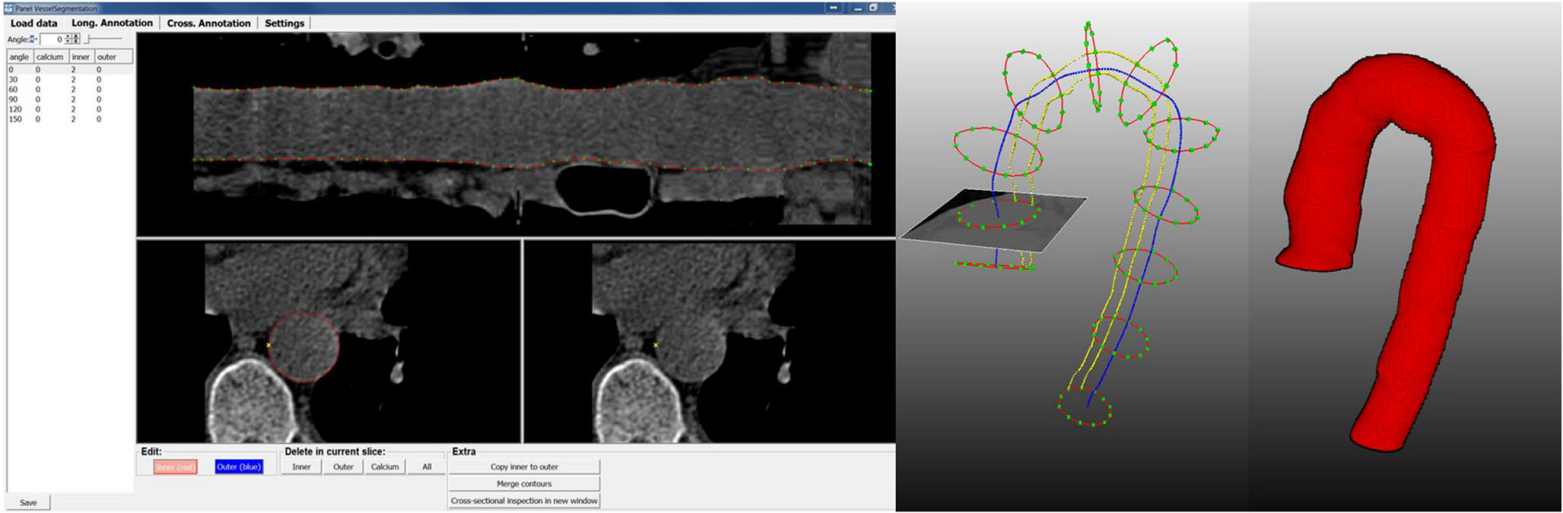
Screenshot of the manual annotation tool (left). Middle image shows two manually drawn longitudinal contours (yellow) and a few cross-sectional contours (red), which are perpendicular to the manual centerline (blue). A cross-sectional slice at the ascending aorta and the corresponding contour is shown as well. The corresponding 3D surface of the aorta is shown on the right

Manual annotations were made using an in-house annotation tool developed in MeVisLab. One hundred CT scans were annotated by a physician (LB) for validation and an additional 25 scans by an experienced observer (ZSG) for method development. The annotation tool was similar to that described previously for carotid artery segmentation in [22]. First, the window level/width was adjusted to 200 HU/600 HU, for all cases. Then, the aortic centerlines were drawn manually using the axial, coronal, and sagittal views, starting from the sinotubular junction of the ascending aorta and ending at the diaphragm level of the descending aorta. Subsequently, the centerlines were checked and modified in reformatted cross-sectional views perpendicular to the drawn centerline. The obtained centerlines were used to generate curved multi-planar reformatted images of the entire aorta, with longitudinal views at six different angles equally spaced every 30° and cross-sectional views every 1 mm along the centerline. Longitudinal contours were drawn manually, whereupon cross-sectional contours were computed using spline interpolation through the intersection points of the longitudinal contours with the cross-sectional planes. Finally, after checking the cross-sectional contours in all cross-sections and adjusting them if required, the contours were converted to a 3D binary image using variational interpolation [23]. An example of manual annotation is shown in Fig. 1.

To manually locate the pulmonary artery bifurcation level, an experienced physician (DB) checked the scans in axial view and annotated the pulmonary artery bifurcation level where the left and right pulmonary arteries and the bifurcation from the pulmonary trunk were all visible.

### Automatic aorta segmentation approach

To extract a full 3D segmentation of the aorta and a landmark point for the pulmonary artery bifurcation level, we applied a combination of image processing techniques. First, to avoid the segmentation to attract to the heart-lung or bone borders, we applied preprocessing as proposed in our previous work [24].

Subsequently, a multi-atlas registration method was applied [25] to localize the aorta, the pulmonary artery trunk, and the left and right pulmonary arteries. In this method, 25 preprocessed CT scans were non-rigidly registered to the scan in which the segmentation was required (target image). From these 25 registered images, the ten with the highest similarity to the target image were selected. The corresponding manual annotations of these ten scans were then deformed and combined using a per voxel majority voting procedure to obtain a coarse initial segmentation of the aorta and pulmonary arteries. The initial segmentation of the pulmonary arteries was then skeletonized, and the slice where main pulmonary artery bifurcates into the left and right pulmonary arteries was extracted as the pulmonary artery bifurcation level. This level is used as the landmark level.

To start tracing the centerline of the aorta, aortic seed points were extracted as the center of mass of the coarse initial aorta segmentation at the axial slice 3 cm beneath the landmark level for the ascending aorta and 6 cm beneath the landmark level for the descending aorta. The aortic centerline was then extracted between these seed points by a minimum cost path tracking algorithm similar to [24]. In this algorithm, the cost function was based on the maximum output of a multi-radius medialness filter in coronal and axial views multiplied with a lumen intensity similarity metric. The centerlines were refined by re-computing the minimum cost path after curved multi-planar reformatting perpendicular to the previous centerline [26]. Failure in the centerline extraction was automatically detected by using the landmark level and the initial pulmonary artery segmentation. Centerlines that did not reach the landmark level or were inside the pulmonary artery segmentation were considered failed extractions and were excluded.

To obtain a first estimate of the aorta, the extracted centerline was dilated using a spherical structuring element with its radius defined by the estimated radius of the aorta obtained from the medialness filter. Subsequently, an optimal surface graph cut segmentation method^1^ [27], initialized by the dilated centerline, was used to accurately extract the surface of the aorta. The parameters for atlas registration, centerline extraction, and graph cut segmentation were tuned to maximize the similarity with manual annotations on 25 CT scans.

### Aortic diameter measurement

**Fig. 2:**
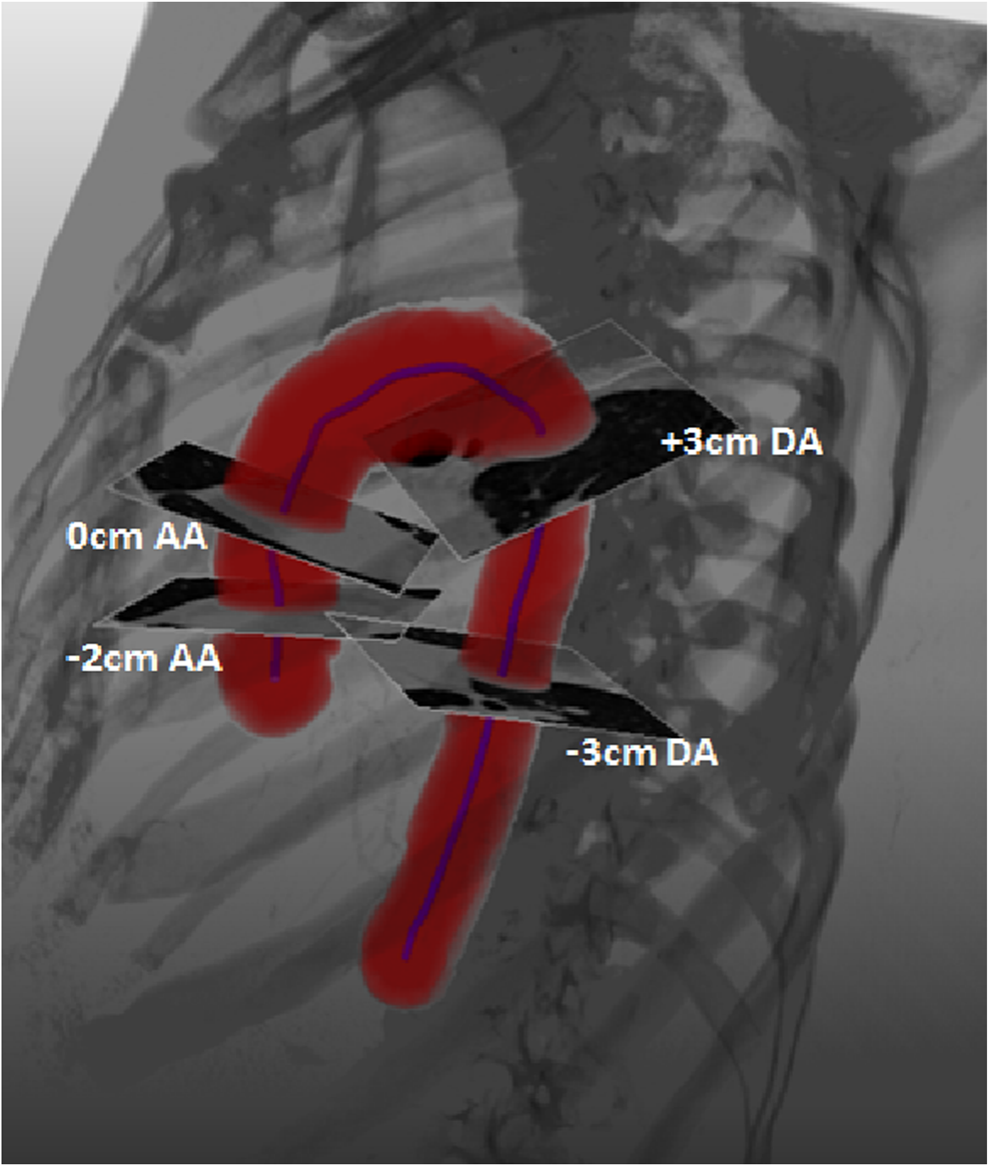
3D automatic segmentation of the aorta and the corresponding automatic centerline showing cross-sections at the ascending aorta at the pulmonary artery bifurcation level (0 cm AA) and at 2 cm below this level (− 2 cm AA) and at the descending aorta at 3 cm above (+ 3 cm DA) and below (− 3 cm DA) the pulmonary artery bifurcation level

Aortic diameters were assessed at multiple, fixed levels relative to the pulmonary artery bifurcation level. Based on the extracted pulmonary bifurcation level, thirteen cross-sectional slices were defined perpendicular to the extracted aortic centerline, located at 1-cm intervals around the bifurcation level from 2 cm below this level to 3 cm above for the ascending aorta and from 3 cm above to 3 cm below this level for the descending aorta. For the ascending aorta, the cross-sectional slice at 3 cm below the pulmonary artery bifurcation level was sometimes in the aortic root below the sinotubular junction which the aorta boundaries at the sinus of Valsalva are very unclear due to the lack of gating and contrast. Therefore, no measurements were performed at this level. Figure 2 shows an example of 3D segmentation with the corresponding centerline and four of the measured cross-sections.

The cross-sectional average aortic diameter at each of the 13 cross-sectional slices was computed from manual and automatic segmentations. For the manual segmentations, diameter measurements were performed perpendicular to the manual centerlines and at levels relative to the manually indicated pulmonary artery bifurcation level. For the automatic segmentations, the automatically extracted centerlines and pulmonary artery bifurcation level were used instead.

### Validation and statistical analysis

The method was validated on 100 CT scans with manual annotations. The segmentation accuracy was assessed by dice similarity coefficient (DSC) and mean surface distance (MSD). DSC [28] measures the degree of spatial overlap of the automatic segmentation with the manual segmentation, and it ranges between 0 and 1, where higher values indicate higher similarity. MSD shows the symmetric mean surface distance in millimeters between the manual and automatic segmentation surfaces, where lower value is better. The agreement between the manual and automatic segmentations was assessed from 3 cm beneath the landmark level at the ascending aorta to 6 cm beneath this level at the descending aorta. DSC, MSD, aortic diameters, and the error in the diameter were expressed as mean ± standard deviation (range).

The error in the extracted landmark level was assessed by the distance between the manually extracted pulmonary artery bifurcation level and the automatically extracted level in millimeters. The aortic centerlines were automatically checked for failed extractions.

The agreement between the manual and automatic diameter measurements was assessed by (1) intra-class correlation (ICC) based on a single-rating, absolute-agreement, two-way mixed-effects model [29]; (2) *R*^2^ Pearson’s correlation; and (3) Bland-Altman analysis.

Repeatability of the method was assessed by comparing the automatically extracted diameters of two scans of 617 subjects with time period of 1 year in between. Within 1 year, changes in aortic diameters are expected to be small, with 0.1–0.2-mm growth per year in a healthy population [3, 30]. All statistical analyses were done in MATLAB.

## Results

**Fig. 3:**
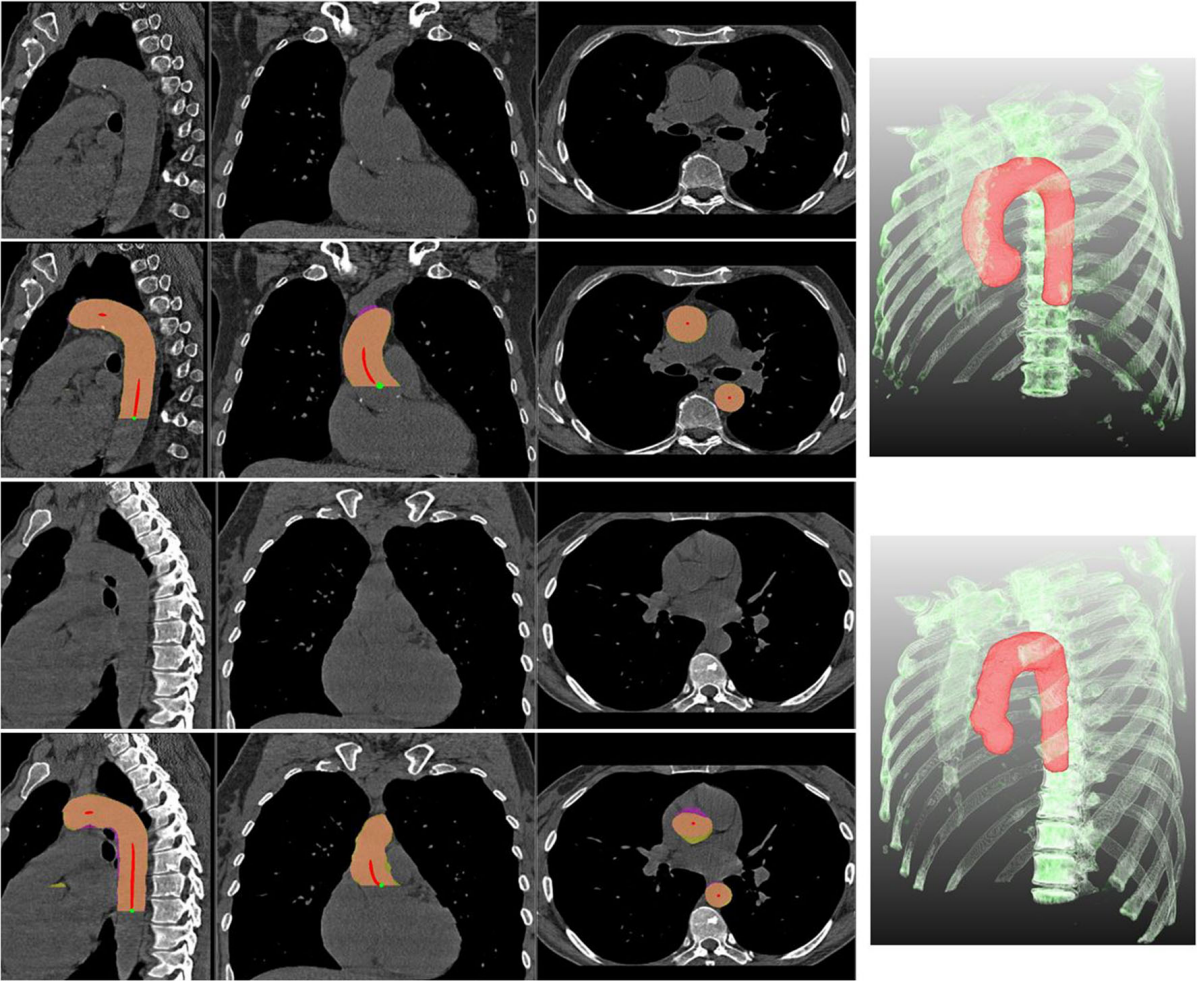
Two samples with the best (top two rows) and the worst (bottom two rows) automatic segmentation results. The columns from left show the sagittal, coronal, and axial views, respectively. The right column shows the 3D visualization of the automatic segmentation in red. First and third rows are the original CT scans, while the second and fourth rows show the CT scan with the overlap of the corresponding manual and automatic segmentations with DSC = 0.96 and MSD = 0.60 mm for the first sample and DSC = 0.92 and MSD = 1.44 mm for the second sample. Orange shows the regions where the manual and automatic segmentations overlap. Magenta is the region included in the automatic segmentation, but not in the manual segmentation, and yellow is the region that is inside the manual segmentation, but not in the automatic segmentation. Centerline points are indicated in red and seed points in green

Figure 3 shows examples of segmentation results. Out of all 1334 CT scans only in two cases, the seed points at the descending aorta were extracted incorrectly. Centerline extraction further failed in seven cases, all of which were easily detected automatically. Average DSC for the entire aorta was 0.95 ± 0.01 (0.92–0.96) and MSD was 0.56 ± 0.08 (0.43–0.93) mm. The mean absolute distance between the manual and automatic landmark level of the pulmonary artery bifurcation was 2.55 ± 1.94 mm, with almost no bias (mean signed distance 0.45 ± 3.18 mm).

**Fig. 4:**
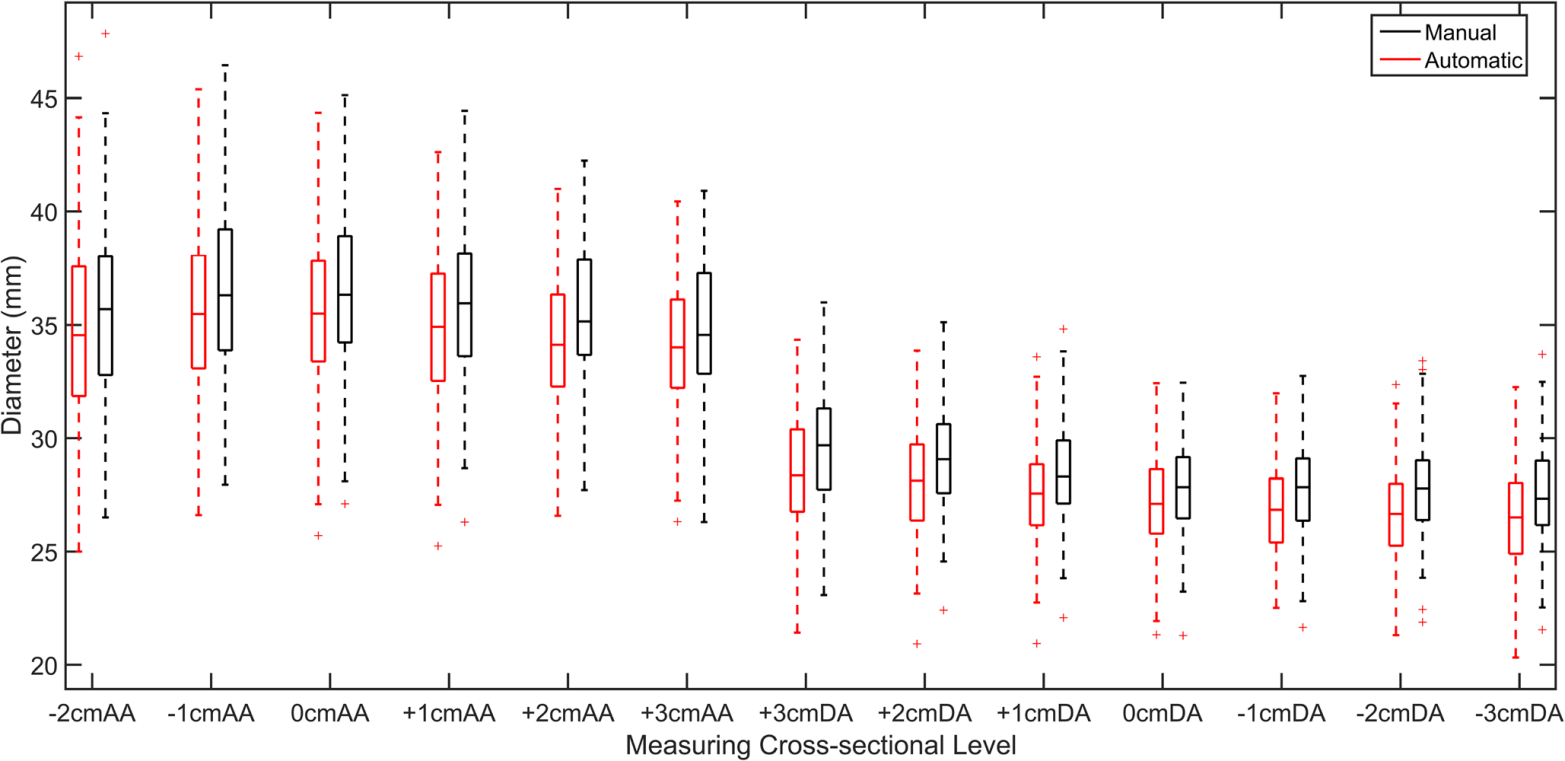
Average manual (black) and automatic (red) diameter per measuring level. From left to right, diameters measured at the different levels along the aorta from 2 cm below the pulmonary artery bifurcation level (0 cm) at the ascending aorta (AA) to 3 cm below the pulmonary artery bifurcation level at the descending aorta (DA)

Box plots for the average manual and automatic diameters for each measuring level are shown in Fig. 4. Diameters measured at the different levels, for men and women separately, are shown in Table 2. High agreement between manually and automatically measured diameters was obtained, with an overall ICC and *R*^2^ Pearson’s correlation of 0.97. The level-wise correlations together with the correlations separated per gender are shown in Table 3 (see supplementary Fig. S2 for scatter plots of each measuring level).

**Table 2:** Average aortic diameters from the automatic and manual segmentations for each measuring level from the 100 CT scans. Values are expressed as mean ± standard deviation

	Measuring level	Female (*n* = 50)		Male (*n* = 50)	
		Automatic	Manual	Automatic	Manual
Ascending aorta	− 2 cm	33.3 ± 3.5	34.2 ± 3.5	36.2 ± 3.9	37.0 ± 3.7
	− 1 cm	34.2 ± 3.2	35.1 ± 3.4	36.9 ± 3.7	37.7 ± 3.6
	0 cm	33.9 ± 3.3	35.0 ± 3.3	36.7 ± 3.5	37.7 ± 3.5
	+ 1 cm	33.5 ± 3.0	34.7 ± 3.2	36.3 ± 3.6	37.4 ± 3.5
	+ 2 cm	33.3 ± 2.7	34.3 ± 2.9	35.4 ± 3.3	36.7 ± 3.2
	+ 3 cm	33.0 ± 2.9	33.4 ± 3.1	35.2 ± 3.0	36.0 ± 3.2
Descending aorta	+ 3 cm	27.6 ± 2.6	28.7 ± 2.5	29.5 ± 2.4	30.8 ± 2.5
	+ 2 cm	27.3 ± 2.5	28.2 ± 2.4	29.0 ± 2.3	30.2 ± 2.1
	+ 1 cm	26.7 ± 2.3	27.5 ± 2.2	28.5 ± 2.1	29.4 ± 2.0
	0 cm	26.3 ± 2.3	27.1 ± 2.2	28.0 ± 2.0	28.9 ± 1.9
	− 1 cm	26.1 ± 2.1	26.9 ± 2.2	27.7 ± 2.1	28.9 ± 1.9
	− 2 cm	25.8 ± 2.2	26.9 ± 2.2	27.6 ± 2.0	28.6 ± 1.8
	− 3 cm	25.5 ± 2.3	26.7 ± 2.2	27.4 ± 2.1	28.3 ± 2.0

0 cm is the pulmonary artery bifurcation level, where minus is level below this level and plus is level above the pulmonary bifurcation level

**Table 3:** ICC and *R*^2^ Pearson’s correlation between the automatic and manual diameters for the 100 CT scans

	Measuring level	ICC (*n* = 100)	*R*^2^ Pearson (*n* = 100)	ICC female (*n* = 50)	ICC male (*n* = 50)
Ascending aorta	− 2 cm	0.93	0.90	0.89	0.94
	− 1 cm	0.94	0.94	0.91	0.95
	0 cm	0.94	0.95	0.92	0.94
	+ 1 cm	0.92	0.94	0.89	0.92
	+ 2 cm	0.92	0.95	0.91	0.90
	+ 3 cm	0.94	0.93	0.94	0.93
Descending aorta	+ 3 cm	0.88	0.92	0.88	0.85
	+ 2 cm	0.88	0.91	0.88	0.84
	+ 1 cm	0.89	0.92	0.88	0.87
	0 cm	0.90	0.93	0.90	0.87
	− 1 cm	0.89	0.94	0.90	0.83
	− 2 cm	0.87	0.93	0.86	0.83
	− 3 cm	0.89	0.95	0.86	0.88

Measuring levels as in Table 2

*ICC* intra-class correlation

**Fig. 5:**
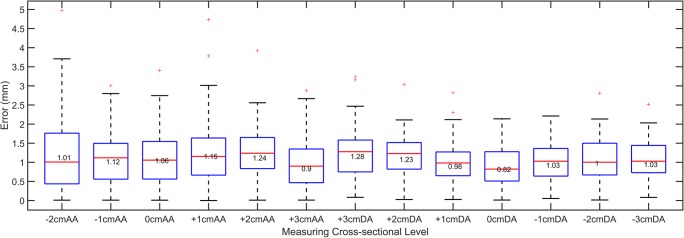
Absolute difference between the aortic diameters obtained from automated and manual 3D segmentations. From left to right, diameters measured at the different levels along the aorta from 2 cm below the pulmonary artery bifurcation level (0 cm) at the ascending aorta (AA) to 3 cm below the pulmonary artery bifurcation level at the descending aorta (DA). The box plot shows the median (red line), interquartile range (boxes), the 99.3% coverage of the data (whiskers), and the outliers (+ symbol)

**Fig. 6:**
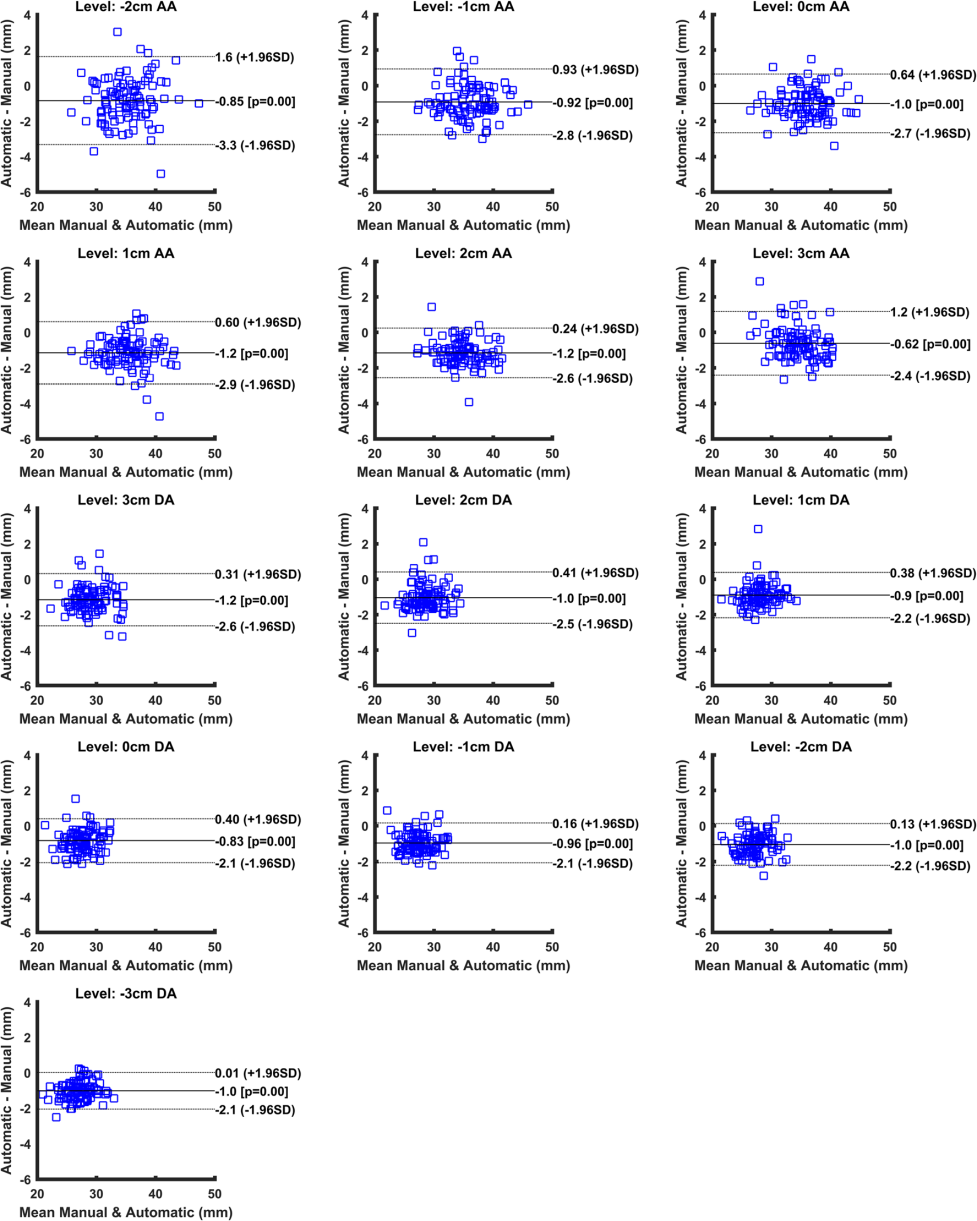
Bland-Altman plots for each measuring level from 2 cm below the pulmonary artery bifurcation in the ascending aorta (AA) until 3 cm below this level in the descending aorta (DA). The measuring level, limits of agreement, and the mean difference are displayed on the plots

An average absolute diameter error of 1.09 ± 0.6 mm between manual and automatic diameters was obtained over all measuring levels, which showed a slight underestimation of the automated measurements compared to manual measurements (mean signed error − 0.97 ± 0.8 mm). As shown in box plots of the level-wise diameter errors in Fig. 5, larger errors (more than 3 mm) were extracted in 8 out of 100 scans. In four cases, a large error occurred due to motion artifacts at the ascending aorta (beneath the landmark level), and in three cases, it occurred at the aortic arch due to branching arteries. In one case, the error was along the entire aorta due to a 6-mm difference between the automatic and manual landmark levels. Bland-Altman plots of manual and automated diameter measurements are given in Fig. 6.

From the 617 subjects used to assess repeatability, 7 subjects had failed centerline or seed point extraction. From the remaining 610 subjects, ICC between the automatic diameters of the scan and rescan of each subject is shown in Table 4. From these 610 subjects, 72 subjects (12%) had an absolute diameter difference larger than 3 mm between the two time points at any of the measuring levels. In 35 out of 72 cases (48.6%), the segmentations appeared visually correct in both time points. In 17 cases of these 35 cases, a 2- or 3-mm difference between the extracted landmark level in one of the time points resulted in big diameter differences at 2 cm below the landmark level at the ascending aorta (in average 3.7 ± 0.5 mm). This is due to the aortic anatomy at the sinotubular junction where the aorta below this level is on average 3 mm larger than above [31]. In 5 out of 35 cases, there was more than 6-mm difference between the extracted landmark levels from the two time points, leading to a diameter measurement at very different levels along the entire aorta being compared (in average 3.4 ± 0.5 mm). The remaining 13 out of 35 cases appeared to have a slightly larger diameter at one of the time points (in average 3.7 ± 0.7 mm), possibly due to the aortic size changes during the cardiac cycle. In 37 out of 72 cases (51.4%), the average diameter difference (3.6 ± 0.6 mm) was due to segmentation error which mainly occurred at the aortic arch which was due to branching arteries, or was at the ascending aorta below the pulmonary artery bifurcation level which was due to heart motion artifacts caused by the non-ECG-gated data.

**Table 4:** Repeatability: ICC between the automatic diameters of the scan and rescan of 610 subjects

	Ascending aorta						Descending aorta						
	− 2 cm	− 1 cm	0 cm	+ 1 cm	+ 2 cm	+ 3 cm	+ 3 cm	+ 2 cm	+ 1 cm	0 cm	− 1 cm	− 2 cm	− 3 cm
ICC	0.91	0.95	0.96	0.96	0.95	0.95	0.94	0.94	0.93	0.94	0.93	0.94	0.94

Measuring levels as in Table 2

*ICC* intra-class correlation

## Discussion

We presented a fully automatic method to segment the thoracic aorta and measure aortic diameters. In our evaluation on 100 non-ECG-gated, non-contrast CT scans, the 3D segmentation algorithm performed well with an average segmentation overlap of 0.95 ± 0.01 and a mean surface distance between manual and automatic segmentations of less than 1 voxel (0.56 mm).

The agreement with diameters obtained from manual segmentations was high, with an overall ICC of 0.97 and an average per-level ICC of 0.91 ± 0.03, which is similar to the agreement reported between observers in [32] (ICC = 0.94). The manual diameters were on average approximately 1 mm larger than automatic diameters. This bias is similar to inter-observer bias reported in [33] for mid-ascending aorta diameter measurement on CTA. Scan-rescan repeatability was high, with an overall ICC of 0.98 and an average per-level ICC of 0.94 ± 0.01.

The mean ascending aorta diameters measured at the pulmonary artery bifurcation level were 36.7 ± 3.5 mm for males and 33.9 ± 3.3 mm for females. These values are similar to those reported by Kalsch et al [3] (37.1 ± 4 mm for males and 34.5 ± 4 mm for females), while they were slightly greater than those reported by Wolak et al [34] (33.5 ± 4 mm for males and 31.4 ± 3 mm for females). These differences may be due to differences in the study populations, CT scan protocol, and measurement approach.

A significant diameter increase of on average 0.11 ± 1.0 mm was measured in repeated scans after 1 year. This agrees well with reported natural yearly aortic diameter growth of 0.1–0.2 mm per year in the healthy population [3, 30]. In 12% of repeat scan pairs (72 subjects), diameter changes larger than 3 mm were observed. In the majority of these cases (44 subjects), large diameter differences occur at the ascending aorta beneath the landmark level which is due to the anatomy and the difficulty of measuring these regions. Due to motion artifacts in the non-ECG-gated scans, segmentation of the proximal part of the aorta including the aortic root is difficult even for experienced radiologists. However, although isolated aortic root aneurysms are seen in patients with Marfan syndrome [9], it is less common than aneurysms of the ascending aorta more distal to the aortic root. Therefore, the aortic root segmentation is less important in our application than the ascending aorta. In the remaining 28 cases, the large diameter difference was either in the aortic arch (15) or in the descending aorta (8) or at multiple locations due to error in the extraction of the pulmonary artery bifurcation level (5). Diameters measured at the aortic arch were visually correct; however, slightly larger diameters were measured at the location of branching arteries. In descending aorta, the large diameter differences were mainly due to segmentation error.

In contrast with our study, in literature, most methods for automatic aorta segmentation were evaluated on CTA in which the aortic lumen is much more clearly visible [10–14]. Few methods were proposed to segment the aorta in non-contrast CT [15–19]. Compared to these previous works, shown in Table 5, our proposed method is evaluated on a larger dataset and shows better performance.

**Table 5:** Performance comparison of methods for the aorta segmentation on non-contrast CT. Values are expressed as mean ± standard deviation

Author [ref. no.]	Evaluation data size	DSC	Jaccard coefficient	MSD (mm)
Kitasaka et al [15]	7 CT	0.93 ± 0.03	–	0.90 ± 0.33
Avila-Montes et al [16]	45 CT	0.84 ± 0.10	0.74 ± 0.13	–
Kurugol et al [17]	45 CT	0.92 ± 0.01	0.85 ± 0.02	0.62 ± 0.09
Isgum et al [18]	29 CT	–	0.78 ± 0.04	–
Xie et al [19]	60 CT	0.93 ± 0.01	–	1.39 ± 0.19
Proposed method	100 CT	0.95 ± 0.01	0.90 ± 0.01	0.56 ± 0.08

*DSC* dice similarity coefficient, *MSD* mean surface distance

We proposed to measure aortic dimensions at fixed intervals with respect to a single anatomical landmark level, the pulmonary artery bifurcation. In clinical practice, multiple anatomical landmarks including locations in the aortic arch are used instead for reporting aortic diameters in CTA [8, 9, 35]. However, consistently extracting these landmarks especially in non-ECG-gated CT is difficult. Moreover, the aorta diameter is poorly defined at the locations of the brachiocephalic artery, left-common carotid artery, and left-subclavian artery. Consistent measurements in the arch require landmark points in between branches that are not affected by this issue; however, detecting such points automatically and robustly in non-contrast CT scans is difficult. Furthermore, aortic dilatation is less common in the arch than in the ascending and descending aorta. Therefore, in this paper, we focus on the ascending and descending aortas which clinically are of more interest. In non-contrast CT, diameters have been mainly measured at the pulmonary artery bifurcation level [2, 3, 34, 36, 37]. The measuring levels used in our study approximately cover the same area used in CTA [8, 9, 35] but are easier to extract reliably in non-contrast and non-ECG-gated CT.

A limitation of our study is that the method was validated only on a relatively healthy screening population. Further investigation would be required to evaluate the performance on abnormal aortic shapes or large aneurysms. However, in all cases with aortic dilatation as indicated in the original radiology reports, the obtained segmentation was correct. In our data, calcification in the aorta was assessed by the Agatston score [38]. Visual inspection of the scans with Agatston score higher than 1500 for the entire aorta (58 out of 742 subjects) showed that the proposed method segmented the calcifications correctly inside the vessel wall in all cases.

The proposed automatic method is a promising technique to accurately and reproducibly assess subtle signs of aorta dilatation in non-ECG-gated, non-contrast CT scans without any human interaction and could be used for efficient screening for aortic dilatation as well as for monitoring of aortic change in clinical practice as part of a comprehensive CT analysis including lung screening.

## Electronic supplementary material

ESM 1 (DOCX 20.1 kb)

ESM 2 Fig. S2 (PNG 797 kb)

## Abbreviations

CT: Computed tomography
CTA: Computed tomography angiography
DSC: Dice similarity coefficient
ICC: Intra-class correlation
MSD: Mean surface distance

## Footnotes

1 Available at https://bitbucket.org/opfront/opfront

## References

[1] Roth GA, Huffman MD, Moran AE (2015). Global and regional patterns in cardiovascular mortality from 1990 to 2013. Circulation.

[2] Itani Y, Watanabe S, Masuda Y (2002). Measurement of aortic diameters and detection of asymptomatic aortic aneurysms in a mass screening program using a mobile helical computed tomography unit. Heart Vessels.

[3] Kälsch H, Lehmann N, Möhlenkamp S (2013). Body-surface adjusted aortic reference diameters for improved identification of patients with thoracic aortic aneurysms: results from the population-based Heinz Nixdorf Recall study. Int J Cardiol.

[4] Mets OM, de Jong PA, Prokop M (2012). Computed tomographic screening for lung cancer. JAMA.

[5] Melvinsdottir IH, Lund SH, Agnarsson BA, Sigvaldason K, Gudbjartsson T, Geirsson A (2016) The incidence and mortality of acute thoracic aortic dissection: results from a whole nation study. Eur J Cardiothorac Surg 50:1111–1117 10.1093/ejcts/ezw235 27334108

[6] Davies RR, Gallo A, Coady MA (2006). Novel measurement of relative aortic size predicts rupture of thoracic aortic aneurysms. Ann Thorac Surg.

[7] Kim JB, Kim K, Lindsay ME (2015). Risk of rupture or dissection in descending thoracic aortic aneurysm. Circulation.

[8] Hiratzka LF, Bakris GL, Beckman JA (2010). 2010 ACCF/AHA/AATS/ACR/ASA/SCA/SCAI/SIR/STS/SVM Guidelines for the diagnosis and management of patients with thoracic aortic disease. A Report of the American College of Cardiology Foundation/American Heart Association Task Force on Practice Guidelines, American Association for Thoracic Surgery, American College of Radiology,American Stroke Association, Society of Cardiovascular Anesthesiologists, Society for Cardiovascular Angiography and Interventions, Society of Interventional Radiology, Society of Thoracic Surgeons,and Society for Vascular Medicine.. J Am Coll Cardiol.

[9] Erbel R, Aboyans V, Boileau C et al (2014) 2014 ESC guidelines on the diagnosis and treatment of aortic diseases. Kardiol Pol 72:1169–252 10.5603/KP.2014.0225 25524604

[10] Gao X, Boccalini S, Kitslaar PH et al (2017) Quantification of aortic annulus in computed tomography angiography: validation of a fully automatic methodology. Eur J Radiol 93:1–8 10.1016/j.ejrad.2017.04.020 28668401

[11] Entezari P, Kino A, Honarmand AR (2013). Analysis of the thoracic aorta using a semi-automated post processing tool. Eur J Radiol.

[12] Elattar MA, Wiegerinck EM, Planken RN (2014). Automatic segmentation of the aortic root in CT angiography of candidate patients for transcatheter aortic valve implantation. Med Biol Eng Comput.

[13] Ecabert O, Peters J, Walker MJ (2011). Segmentation of the heart and great vessels in CT images using a model-based adaptation framework. Med Image Anal.

[14] Biesdorf A, Rohr K, Feng D (2012). Segmentation and quantification of the aortic arch using joint 3D model-based segmentation and elastic image registration. Med Image Anal.

[15] Kitasaka T, Mori K, Hasegawa J, Toriwaki J, Katada K (2002) Automated extraction of aorta and pulmonary artery in mediastinum from 3D chest X-ray CT images without contrast medium. Proc. SPIE 4684, Medical Imaging 2002: Image Processing 10.1117/12.467116

[16] Avila-Montes OC, Kurkure U, Nakazato R, Berman DS, Dey D, Kakadiaris IA (2013) Segmentation of the thoracic aorta in noncontrast cardiac CT images. IEEE J Biomed Health Inform 17:936–949 10.1109/JBHI.2013.2269292 25055373

[17] Kurugol S, Come CE, Diaz AA (2015). Automated quantitative 3D analysis of aorta size, morphology, and mural calcification distributions. Med Phys.

[18] Isgum I, Staring M, Rutten A, Prokop M, Viergever MA, van Ginneken B (2009) Multi-atlas-based segmentation with local decision fusion-application to cardiac and aortic segmentation in CT scans. IEEE Trans Med Imaging 28:1000–1010 10.1109/TMI.2008.2011480 19131298

[19] Xie Y, Padgett J, Biancardi AM, Reeves AP (2014). Automated aorta segmentation in low-dose chest CT images. Int J Comput Assist Radiol Surg.

[20] Pedersen JH, Ashraf H, Dirksen A (2009). The Danish randomized lung cancer CT screening trial--overall design and results of the prevalence round. J Thorac Oncol.

[21] Wille MM, Dirksen A, Ashraf H (2016). Results of the randomized Danish Lung Cancer Screening Trial with focus on high-risk profiling. Am J Respir Crit Care Med.

[22] Hameeteman K, Zuluaga MA, Freiman M (2011). Evaluation framework for carotid bifurcation lumen segmentation and stenosis grading. Med Image Anal.

[23] Heckel F, Konrad O, Karl H, Peitgen H (2013). Interactive 3D medical image segmentation with energy-minimizing implicit functions. Comput Graph.

[24] Sedghi Z, de Bruijne M, Arias AM, Pedersen JH (2018) Aorta and pulmonary artery segmentation using optimal surface graph cuts in non-contrast CT. SPIE 10574, Medical Imaging 2018: Image Processing

[25] Kirişli HA, Schaap M, Klein S (2010). Evaluation of a multi-atlas based method for segmentation of cardiac CTA data: a large-scale, multicenter, and multivendor study. Med Phys.

[26] Tang H, van Walsum T, van Onkelen RS (2012). Semiautomatic carotid lumen segmentation for quantification of lumen geometry in multispectral MRI. Med Image Anal.

[27] Petersen J, Nielsen M, Lo P (2014). Optimal surface segmentation using flow lines to quantify airway abnormalities in chronic obstructive pulmonary disease. Med Image Anal.

[28] Dice LR (1945). Measures of the amount of ecologic association between species. Ecology.

[29] Koo TK, Li MY (2016). A guideline of selecting and reporting intraclass correlation coefficients for reliability research. J Chiropr Med.

[30] Davies RR, Goldstein LJ, Coady MA (2002). Yearly rupture or dissection rates for thoracic aortic aneurysms: simple prediction based on size. Ann Thorac Surg.

[31] Vriz O, Driussi C, Bettio M, Ferrara F, D'Andrea A, Bossone E (2013) Aortic root dimensions and stiffness in healthy subjects. Am J Cardiol 112:1224–1229 10.1016/j.amjcard.2013.05.068 23871268

[32] Terzikhan N, Bos D, Lahousse L et al (2017) Pulmonary artery to aorta ratio and risk of all-cause mortality in the general population: the Rotterdam Study. Eur Respir J 49 10.1183/13993003.02168-2016 28619955

[33] Quint LE, Liu PS, Booher AM, Watcharotone K, Myles JD (2013) Proximal thoracic aortic diameter measurements at CT: repeatability and reproducibility according to measurement method. Int J Cardiovasc Imaging 29:479–488 10.1007/s10554-012-0102-9 PMC3529204 22864960

[34] Wolak A, Gransar H, Thomson LE (2008). Aortic size assessment by noncontrast cardiac computed tomography: normal limits by age, gender, and body surface area. JACC Cardiovasc Imaging.

[35] Hager A, Kaemmerer H, Rapp-Bernhardt U (2002). Diameters of the thoracic aorta throughout life as measured with helical computed tomography. J Thorac Cardiovasc Surg.

[36] Mao SS, Ahmadi N, Shah B (2008). Normal thoracic aorta diameter on cardiac computed tomography in healthy asymptomatic adults: impact of age and gender 1. Acad Radiol.

[37] Lin FY, Devereux RB, Roman MJ (2008). Assessment of the thoracic aorta by multidetector computed tomography: age- and sex-specific reference values in adults without evident cardiovascular disease. J Cardiovasc Comput Tomogr.

[38] Rasmussen T, Køber L, Pedersen JH (2013). Relationship between chronic obstructive pulmonary disease and subclinical coronary artery disease in long-term smokers. Eur Heart J Cardiovasc Imaging.

