# Supplementary material for: Automated 3D segmentation and diameter measurement of the thoracic aorta on non-contrast enhanced CT
Source: Eur Radiol. 2019 Jan 23;29(9):4613–23. doi: 10.1007/s00330-018-5931-z (PMC6682850; doi:10.1007/s00330-018-5931-z)
Supplement: Supplementary file 1 — (DOCX 20.1 kb) [file 330_2018_5931_MOESM1_ESM.docx]

# Supplementary Material

Supplementary Table S1. Clinical characteristics of the data in all three sets. Values are expressed as mean ± standard deviation and (range).Supplementary

|  | **method development (n=25)** | | **validation (n=100)** | | **repeatability (n=617)** | |
| --- | --- | --- | --- | --- | --- | --- |
|  | **Male** | **Female** | **Male** | **Female** | **Male** | **Female** |
| **n** | 12 | 13 | 50 | 50 | 378 | 239 |
| **Age, years** | 57.4±4.0 (51.3-67.8) | 58.3±5.2 (51.5-67.8) | 58.5±5.4 (50.0-70.0) | 58.3±4.8 (50.0-70.0) | 58.9±5.2 (49.3-71.0) | 58 ±5.3 (49.8-70.9) |
| **Weight, kg** | 82.3±10.4 (66-102) | 75.8±9.2 (60-90) | 84.0±12.0 (60-120) | 67.6±12.2 (48-103) | 83.9±12.8 (57-130) | 68.6±12.6 (42-126) |
| **Height, cm** | 177.1±5.0 (167-185) | 167.6±6.3 (158-180) | 179.8±6.3 (163-195) | 167.0±6.1 (155-179) | 179.4±6.5 (163-200) | 166.7±6.1 (150-182) |
| **BMI** | 26.3±3.5 (23.3-33.4) | 27.1±3.9 (20.1-35.2) | 26.0±3.6 (18.7–37.0) | 24.3±4.7 (16.2-41.3) | 26.1±3.7 (17.3-40.9) | 24.7±4.1 (16.4-41.2) |
| **AS at AA & Arch** | 50.4±126.4 (0-435) | 259.1±425.4 (0-1298) | 231.3±416.7 (0-2190) | 193.3±274.4 (0-1128) | 304.3±749.6 (0-6492) | 342.4±742.7 (0-5563) |
| **AS at DA** | 53.3±99.1 (0-256) | 37.8±98.7 (0-336) | 53.5±116.2 (0-483) | 81.4±316.4 (0-2139) | 166.7±696.2 (0-9705) | 224.1± 692.2 (0-5261) |
| n= number of participants; AS= Agatston Score; AA=Ascending Aorta; DA=Descending Aorta | | | | | | |
