# Supplementary figures and images for: Automated 3D segmentation and diameter measurement of the thoracic aorta on non-contrast enhanced CT
Source: Eur Radiol. 2019 Jan 23;29(9):4613–23. doi: 10.1007/s00330-018-5931-z (PMC6682850; doi:10.1007/s00330-018-5931-z)

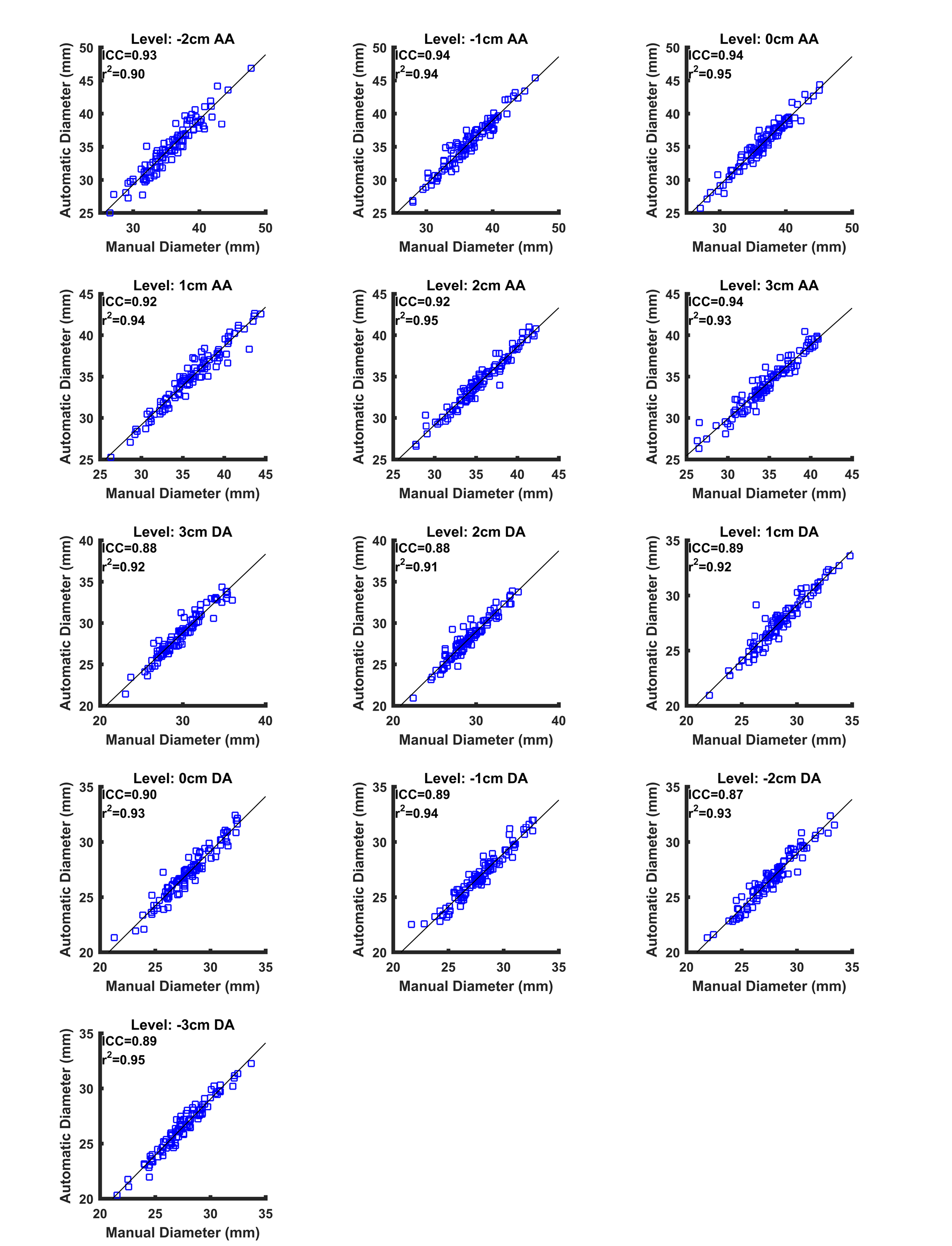

Supplement: Supplementary file 2 — Fig. S2 (PNG 797 kb) [file 330_2018_5931_MOESM2_ESM.png]
